# Fecal Microbiota Transplantation is a Promising Switch Therapy for Patients with Prior Failure of Infliximab in Crohn’s Disease

**DOI:** 10.3389/fphar.2021.658087

**Published:** 2021-05-17

**Authors:** Qianqian Li, Xiao Ding, Yujie Liu, Cicilia Marcella, Min Dai, Ting Zhang, Jianling Bai, Liyuan Xiang, Quan Wen, Bota Cui, Faming Zhang

**Affiliations:** ^1^ Medical Center for Digestive Diseases, The Second Affiliated Hospital of Nanjing Medical University, Nanjing, China; ^2^ Department of Medicine and Therapeutics, The Chinese University of Hong Kong, Hong Kong, China; ^3^ Department of Biostatistics, School of Public Health, Nanjing Medical University, Nanjing, China; ^4^ Key Lab of Holistic Integrative Enterology, Nanjing Medical University, Nanjing, China; ^5^ National Clinical Research Center for Digestive Diseases, Xi’an, China

**Keywords:** fecal microbiota transplant, anti-tumor necrosis factor, infliximab, washed microbiota transplantation, crohn’s disease

## Abstract

**Background:** How to handle patients with anti-tumor necrosis factor (anti-TNF) failure was a common challenge to clinicians in Crohn’s disease (CD). The present study is dedicated to clarifying whether fecal microbiota transplantation (FMT) could be a switch therapy for patients with prior failure of infiiximab (IFX) in CD in a long-term observation.

**Methods:** Thirty-six patients with CD who had prior failure of IFX were recruited from January 2013 to December 2019. The “one-hour FMT protocol” was followed in all patients. All patients received the first course of FMT through gastroscopy or mid-gut transendoscopic enteral tubing. After April 2014, the methodology of FMT was coined as washed microbiota transplantation (WMT), substituting for the manual methods, which is dependent on the automatic microbiota purification system and the washing process. The primary endpoint of this study was the clinical remission at one month and one year after FMT. The secondary endpoint was the safety of FMT in the short and long term, and clinical factors as predictors for long-term efficacy of FMT. Clinical factors as independent predictors of efficacy from FMT were isolated using univariable and multivariable logistic regression analysis.

**Results:** There was no significant difference in the rates of clinical response and remission between IFX treatment stage and FMT treatment stage (at one month, three months and six months after administration) (*p* > 0.05). Compared with those of 19 patients who achieved clinical remission at one month after FMT, the rates of clinical relapse were significantly higher in 18 patients who achieved clinical remission at one month after IFX [log-rank test *p* = 0.0009 HR = 3.081 (95% CI 1.43–6.639)]. Multivariate analysis revealed that the gender of donor (95% CI: 0.001–0.72; *p* = 0.031) was an independent predictor of efficacy at one year after FMT. No serious adverse events (AEs) associated with FMT were observed during and after FMT. The rate of AEs was significantly lower in group FMT than that in group IFX (*p* = 0.002).

**Conclusion:** The present findings first time provided the evidence for clinicians to consider FMT into practice as an alternative switch therapy for patients with prior loss of response or intolerance to IFX in CD.

**Clinical Trial Registration:** https://clinicaltrials.gov, identifier NCT01793831

## Introduction

Crohn’s disease (CD) is a chronic relapsing-remitting inflammatory condition that can affect the entire gastrointestinal tract, leading to a disease course of irreversible bowel damage complicated by strictures and fistulas. The characteristic symptoms of CD include diarrhea, abdominal pain, weight loss and fatigue, which lead to a persistently negative impact on patients’ well-being and quality of life (Baumgart and Sandborn, 2007; Thia et al., 2010; Becker et al., 2015).

Over the past two decades, infliximab (IFX), a monoclonal IgG1 anti-tumor necrosis factor (TNF) antibodies has proven to be effective in patients with moderate-to-severe CD refractory to conventional therapies (Hanauer et al., 2002; Colombel et al., 2007; Terdiman et al., 2013; Gagniere et al., 2015; Gionchetti et al., 2017). However, approximately 30% of patients with inflammatory bowel disease (IBD) fail to respond to anti-TNF, this condition is often known as primary non-response (PNR) (when a patient does not respond to an induction regimen of the biological agent). In addition, the development of infusion reactions manifested as intolerance, including acute or delayed reactions, and secondary loss of response (SLOR) (when a patient has initially responded to a biological agent but loses response over time) in almost 50% of patients (Gisbert and Panes, 2009; Billioud et al., 2011; Fine et al., 2019; Papamichael et al., 2020) are both the limitations of anti-TNF when it serves as maintenance therapy. Importantly, patients with primary failure of IFX often had no response to the second-line biologic drugs, thus leading to prolonged disease activity (Roblin et al., 2018; Roblin et al., 2020). So far, there is no international consensus on how to handle IBD patients with failure of anti-TNF (Allez et al., 2010; Papamichael et al., 2015; Buhl et al., 2017). Appropriately choosing other therapies after anti-TNF failure was a common challenge to clinicians, yet little is known about the prognosis after the failure in the long term (Hanauer et al., 2002; Allez et al., 2010).

Increasing evidence suggests that fecal microbiota transplantation (FMT) is a promising approach for CD by remodeling the construction of gut microbiota in a short-term observation and with a low incidence of serious AEs (Cui et al., 2015b; Kong et al., 2020; Sokol et al., 2020). Our previous study by He et al. revealed that patients with CD could achieve significant long-term relief in abdominal pain followed by FMT. We also ascertained that three months after the initial course of FMT was recommended as the optimal timing for the second course of FMT in patients with CD (Cui et al., 2015b; Li et al., 2019). Additionally, we first analyzed the long-term outcomes in the largest cohort (174 patients with CD who underwent FMT). The findings encouraged the clinicians to re-evaluate the therapeutic value of FMT in CD by the targets, including abdominal pain, hematochezia, fever and diarrhea, beyond the traditional evaluations that use Harvey-Bradshaw index (HBI) or Crohn’s disease Activity Index (Xiang et al., 2020). However, none of the studies evaluated the efficacy and safety of FMT for CD patients with prior loss of response or intolerance to IFX in a long-term observation.

This study aimed to compare the efficacy and safety between IFX treatment stage and FMT treatment stage in the patients with CD from the time of longitudinal perspective; and explore clinical factors as the predictors of the short-term and long-term efficacy of these patients followed by FMT. Importantly, we were dedicated to identifying whether FMT could be a promising switch therapy for patients with CD who with prior loss of response or intolerance to IFX in a long-term observation.

## Materials and Methods

### Study Design

This is a prospectively and retrospectively observational study evaluating patients with CD from January 2013 to December 2019, which is a part of the clinical trial (NCT01793831). All eligible subjects provided written informed consents before participation. Inclusion criteria were: patients who were diagnosed as CD by a combination of typical clinical symptoms, endoscopic, and histological criteria for at least 6 months; patients with active CD (HBI ≥ 5); patients who failed to achieve satisfactory efficacy from the previous therapies and had prior loss of response or intolerance to IFX. Patients were excluded if aged < 14 years, accompanied by other severe diseases, including other intestinal diseases, e.g., *Clostridioides difficile* infection, malignant neoplasm, cardiopulmonary failure, and serious liver and kidney disease, and follow-up less than 3 months.

The follow-up was performed at one month, three months, six months and every three months after the first FMT and terminal follow-up was completed on April 1, 2020. The primary endpoint was the clinical remission at one month and one year after FMT. The secondary endpoint was the safety of FMT in the short and long term and clinical factors as predictors for long-term efficacy of FMT. The failure of IFX included that patients had PNR, SLOR or intolerance to IFX. Before FMT, the baseline patient characteristics were recorded, which included age, gender, age of diagnosis, age of onset, disease duration, current smoker, previous treatment and history of previous surgery related to CD. Baseline HBI, disease activity, disease location and disease phenotype of patients were assessed. The concomitant medications after FMT and detailed process of FMT were noted, including delivering route, form of bacterial suspension, preparation of fecal microbiota, frequency of FMT (single or multiple FMTs) and age and gender of donor. In addition, details of IFX treatment stage were also recorded, including characteristics of patients at baseline of IFX, history of surgery and previous medication before IFX, frequency and dose of IFX (Table 1). Moreover, the short and long-term efficacy, and safety of IFX were also evaluated (Table 1).

**TABLE 1:** The details of infliximab before fecal microbiota transplantation.

Pt	Outcomes of IFX	HBI pre-IFX	Age at IFX (yr)	Duration of disease pre-IFX (yr)	Frequency of IFX infusion	Dose of IFX (≥5 mg/kg)	Perianal disease pre-IFX	Concomitant medications* after initial IFX	Efficacy (1 month)	Maintaining time (mo)	AEs	Time between final IFX and FMT (mo)
1	PNR	9	30	6	6	Yes	Anal fistula	Corticosteroid	No response	0	No	42
2	Intolerance	15	22	6	2	Yes	None	EEN	Response	2	Yes	1
3	SLOR	7	24	6	9	Yes	Anal fistula	AZA	Remission	13	No	36
4	Intolerance	5	23	1	6	Yes	None	AZA	Remission	9	Yes	12
5	Intolerance	25	24	1	2	Yes	None	Corticosteroid/EEN	No response	0	Yes	12
6	SLOR	7	18	8	6	No	None	Corticosteroid/EEN	No response	0	Yes	90
7	Intolerance	5	34	1	3	Yes	Abscess	Corticosteroid	Remission	1	Yes	1
8	SLOR	11	22	6	6	Yes	None	Corticosteroid	No response	0	No	12
9	Intolerance	9	28	1	3	Yes	None	None	No response	0	Yes	1
10	PNR	6	46	11	6	Yes	Anal fistula	None	No response	0	No	18
11	SLOR	19	28	1	6	Yes	None	AZA	Remission	13	Yes	90
12	SLOR	15	33	1	4	Yes	None	None	Remission	4	No	12
13	SLOR	15	30	7	15	Yes	Anal fistula	AZA	Remission	24	No	24
14	SLOR	11	34	18	9	No	Anal fistula	None	Remission	13	Yes	18
15	PNR	9	24	7	9	No	None	AZA	No response	0	No	12
16	SLOR	5	43	8	14	No	None	None	Remission	22	Yes	24
17	Intolerance	13	14	2	6	Yes	None	None	No response	0	Yes	1
18	PNR	8	15	1	15	Yes	None	None	No response	0	No	2
19	Intolerance	12	19	1	2	Yes	Anal fistula	None	Remission	7	Yes	7
20	PNR	6	12	1	4	No	None	None	No response	0	No	6
21	SLOR	6	37	4	5	Yes	None	None	Remission	25	No	24
22	SLOR	11	32	8	10	Yes	Anal fistula	AZA	Response	15	No	18
23	SLOR	13	20	1	8	Yes	None	None	Remission	11	No	48
24	Intolerance	5	23	1	6	Yes	None	None	Remission	7	Yes	6
25	SLOR	9	28	2	9	Yes	None	None	Remission	13	No	12
26	SLOR	12	39	9	8	Yes	None	None	Response	11	No	12
27	SLOR	6	26	1	6	Yes	Anal fistula	None	Remission	7	Yes	6
28	SLOR	9	28	6	6	Yes	Anal fistula	AZA	Remission	10	No	12
29	Intolerance	5	39	7	15	Yes	None	None	Remission	24	Yes	24
30	SLOR	15	25	1	19	Yes	None	AZA	Remission	31	No	6
31	Intolerance	13	34	6	6	No	None	None	No response	0	Yes	36
32	SLOR	15	31	8	10	No	None	None	Remission	39	No	48
**#**	NA	9.0 (6.0–13.0)	28.0 (22.3–33.8)	6.0 (1.0–8.0)	6.0 (5.3–9.0)	NA	NA	NA	NA	7.0 (0–13)	NA	12.0 (6.0–24.0)

Pt, patients; IFX, infliximab; HBI, Harvey-Bradshaw index; *5-ASA was the concomitant medications after initial IFX in 75% (24/32) of patients; mo, month; AEs, adverse events; FMT, fecal microbiota transplantation; PNR, primary non-response; SLOR, secondary lose of response; EEN, exclusive enteral nutrition; AZA, azathioprine; Line #, median (IQR); NA, not applicable.

5-Aminosalicylic acid was a sustained treatment before and after FMT if patients had no allergic response. Azathioprine or thalidomide were possibly administered during the tapering off steroids or as maintenance therapies after FMT. Patients were supported by exclusive enteral nutrition (EEN) for at least one month if they had malnutrition, severe stricture or intestinal fistula. Usage of probiotics was prohibited after FMT. Antibiotics were not recommended to be used at random before conducting and communicating with the clinicians.

### Donor Screening and FMT Procedure

Patients could self-identify their relatives or friends as donors at the early stage of the study. The unrelated universal donors aged from 6 to 24 years old were from Chinese fecal microbiota bank (fmtBank) and they were selected by strict screening criteria according to our previous reports (Cui et al., 2015b; Ding et al., 2019; Li et al., 2020b). After April 2014, the methodology of FMT was coined as washed microbiota transplantation (WMT), substituting for the manual methods, which is dependent on the automatic washing process and related delivering consideration. Additionally, WMT is demonstrated to be safer, more precise and more quality-controllable than the crude FMT by manual (Zhang et al., 2020). The methodology of WMT was released by a consensus panel of the FMT-standardization Study group in 2019 (Fecal Microbiota Transplantation-standardization Study, 2020). From the process of feces defecation until the fresh bacteria infused into the intestinal tract of patients should be done within one hour, which was regarded as a “one-hour FMT protocol” (Zhang et al., 2018).

Single FMT through gastroscopy to transfer microbiota suspension into the distal duodenum under anesthesia was performed from 2013 to 2019 in most patients with CD in this study. In order to prevent the refluxing of microbiota liquid and inhibit gastric acid secretion, patients were given metoclopramide 10 mg by intramuscular injection and proton pump inhibitor intravenously at least one hour before FMT. With the increasing needs for frequent FMTs (multiple FMTs), two types of transendoscopic enteral tubing (TET) (FMT Medical, Nanjing, China), including colonic TET and mid-gut TET, were used for delivering washed microbiota suspension in practice since 2014 (Peng et al., 2016; Long et al., 2018). In the present study, mid-gut TET also was applied for EEN.

### Efficacy and Safety Assessment

The clinical efficacy of all patients was assessed at one month, three months, six months and every three months after the initial IFX and FMT. HBI was used to evaluate the clinical efficacy of IFX and FMT. Clinical response was defined as HBI ≤ 4 or decreased HBI score > 3 from baseline, and clinical remission was defined as HBI ≤ 4 (Xiang et al., 2020). Relapse was defined as HBI ≥ 5 after achieving clinical remission and increased HBI ≥ 2 after achieving clinical response. Common Terminology Criteria for Adverse Events (version 5.0) was applied to describe the intensity and relativity of AE with IFX and FMT. Only IFX and FMT-attributed AEs were reported in the study, including definitely, probably, and possibly related AEs. Acute infusion reaction (AIR) was defined by the occurrence of any AE during or within the first 24 h after IFX infusion. Delayed hypersensitivity reactions (DHR) was considered as AEs occurring 24 h later after IFX administration (Zabana et al., 2010).

### Statistical Analysis

The data were assessed by SPSS Statistics (SPSS Inc. Chicago, IL, United States) or GraphPad (version 5; GraphPad Software, San Diego, CA, United States). Normally distributed continuous data were expressed with mean ± standard deviation (SD) and abnormally distributed continuous data were expressed with median with interquartile ranges (IQR). The clinical efficacy and safety between IFX treatment stage and FMT treatment stage were compared by Fisher’s exact test. Rates of relapse were compared using Kaplan-Meier curves and log-rank statistics. Clinical factors as independent predictors of efficacy from FMT were isolated using multivariable logistic regression analysis, and receiver operating characteristic curve (ROC) analysis was used to examine the predictor model. *p* < 0.05 was considered statistically significant.

## Results

### Study Population

**FIGURE 1:**
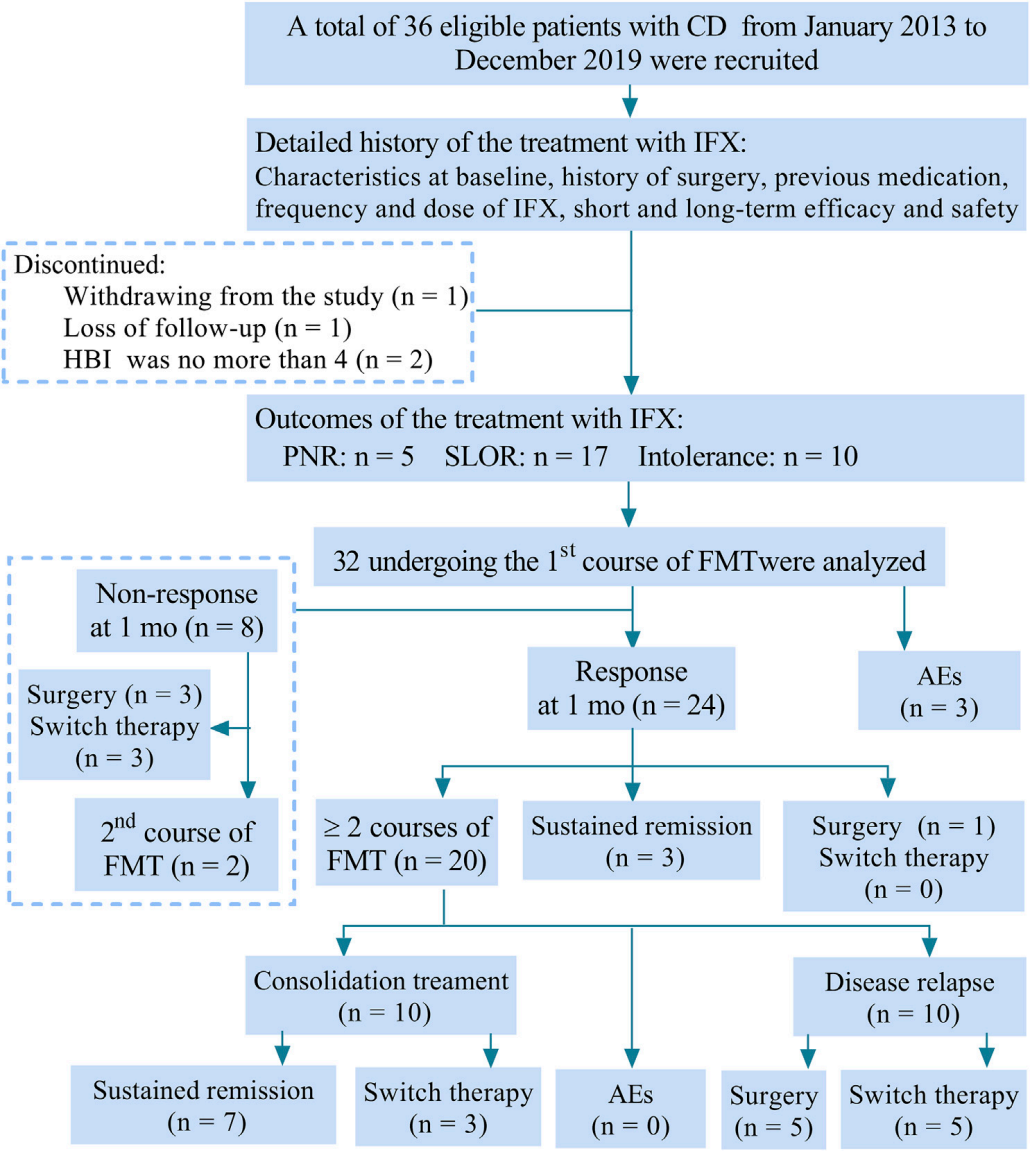
Flow chart of the study. CD, Crohn’s disease; IFX, infliximab; HBI, Harvey-Bradshaw index; PNR, primary non-response; SLOR, secondary loss of response; FMT, fecal microbiota transplantation; mo, month; AEs, adverse events.

This single-center study included 36 eligible patients with prior IFX failure who underwent the first course of FMT from January 2013 to December 2019 (Figure 1). Four patients were excluded from being analyzed for withdrawing from the study (n = 1), loss of follow-up (n = 1) and HBI ≤ 4 (n = 2). Clinical data on IFX and FMT of 32 patients were collected at baseline during the procedure of FMT and long-term follow-up.

Among the 32 patients included in the analysis, 15.6% (5/32) of patients had PNR, 53.1% (17/32) had SLOR and 31.3% (10/32) were intolerant to IFX. Meanwhile, 75% (24/32) of patients achieved clinical response at one month after FMT, in which 20 patients underwent more than one course of FMT, including 10 patients with disease relapse before the second course of FMT and 10 patients with the second course of FMT before disease relapse for consolidation. In a total of 32 patients, 31.3% (10/32) had sustained remission until April 1, 2020 (the terminal point of follow-up), including 7 patients undergoing more than one course of FMT.

### Patient Characteristics

The characteristics of all 32 patients who had a history of IFX failure were shown in Table 2. The mean follow-up was 50.9 ± 24.4 months. The mean age of these patients was 29.8 ± 8.4 years old and the mean disease duration was 6.5 (3.0–10.0) years. 68.8% (22/32) of patients were male. 59.4% (19/32) of patients were with moderate and severe CD. Before the initial course of FMT, those patients had various medication regimens: 96.9% (31/32) were on 5-ASA, 59.4% (19/32) were on corticosteroids, 65.6% (21/32) were on azathioprine, 31.2% (10/32) were on enteral nutrition and 100% (32/32) were on IFX. After the initial course of FMT, 75% (24/32) had 5-ASA, only 9.4% (3/32) patients had corticosteroids and 15.6% (5/32) patients had azathioprine. In addition, 25% (8/32) patients had thalidomide and 40.6% (13/32) patients had EEN. 65.6% (21/32) of patients underwent FMT through gastroscopy. 84.4% (27/32) patients underwent single FMT in the first course of FMT. Most of the microbiota suspension which was transferred into the gut of patients was fresh (87.5%, 28/32), and 68.7% (22/32) of those were prepared by the method of WMT (Zhang et al., 2020).

**TABLE 2:** Characteristics of included patients.

Characteristic	N = 32
Age at FMT, (years, mean ± SD)	29.8 ± 8.4
Gender, male, n (%)	22 (68.8)
Age of diagnosis, (years, mean ± SD)	24.4 ± 6.6
Time from diagnosis to first FMT, (years, median (IQR))	5.5 (2.0–9.8)
Age of onset, (years, mean ± SD)	22.7 ± 7.2
Duration of disease, (years, median (IQR))	6.5 (3.0–10.0)
Follow-up, (months, mean ± SD)	50.9 ± 24.4
Time between final IFX and FMT, (months, median (IQR))	12.0 (6.0–24.0)
Current smoker, n (%)	5 (15.6)
Corticosteroid-dependent, n (%)	2 (6.3)
Baseline HBI, median (IQR)	9.0 (6.0–13.0)
Baseline disease activity, n (%)	
Mild (5–7)	13 (40.6)
Moderate (8–16)	17 (53.1)
Severe (≥17)	2 (6.3)
Age at diagnosis, year, n (%)	
A1 (<17)	5 (15.6)
A2 (17–40)	27 (84.4)
A3 (>40)	0 (0)
Disease location, n (%)	
Ileum (L1)	5 (15.6)
Colon (L2)	6 (18.7)
Ileocolic (L3)	18 (56.3)
Upper tract (L4) + (L1/L2/L3)	3 (9.4)
Phenotype, n (%)	
Inflammatory (B1)	11 (34.4)
Stricturing (B2)	14 (43.7)
Penetrating (B3)	7 (21.9)
Perianal disease (*p*), n (%)	5 (15.6)
Previous therapy, n (%)	
5-ASA	31 (96.9)
Corticosteroid	19 (59.4)
Azathioprine	21 (65.6)
Enteral nutrition	10 (31.3)
infliximab	32 (100)
Concomitant medications, n (%)	
5-ASA	24 (75)
Corticosteroid	3 (9.4)
Azathioprine	5 (15.6)
Thalidomide	8 (25)
EEN	13 (40.6)
Previous surgery related to CD, n (%)	
Perianal	9 (28.1)
Resection	5 (15.6)
Colectomy	2 (6.3)
Ostomy	2 (6.3)
Delivering route, n (%)	
Gastroscopy	21 (65.6)
Mid-gut TET	7 (21.9)
Frequency of the 1st FMT, n (%)	
Single	27 (84.4)
Multiple (≥2)	5 (15.6)
Form of bacterial suspension, n (%)	
Fresh	28 (87.5)
Frozen	4 (12.5)
Preparation of fecal microbiota	
Manual	10 (31.3)
Automatic	22 (68.7)
Age of donor, (years, median (IQR))	17.5 (13–23)
Gender of donor, n (%)	
Male	12 (37.5)
Female	20 (62.5)

IQR, interquartile range; IFX, infliximab; HBI, Harvey–Bradshaw Index; FMT, fecal microbiota transplantation; CRP, C-reactive protein; TET, transendoscopic enteral tubing; EEN, exclusive enteral nutrition; CRP, C-reactive protein.

### Maintaining Time of the Efficacy

**FIGURE 2:**
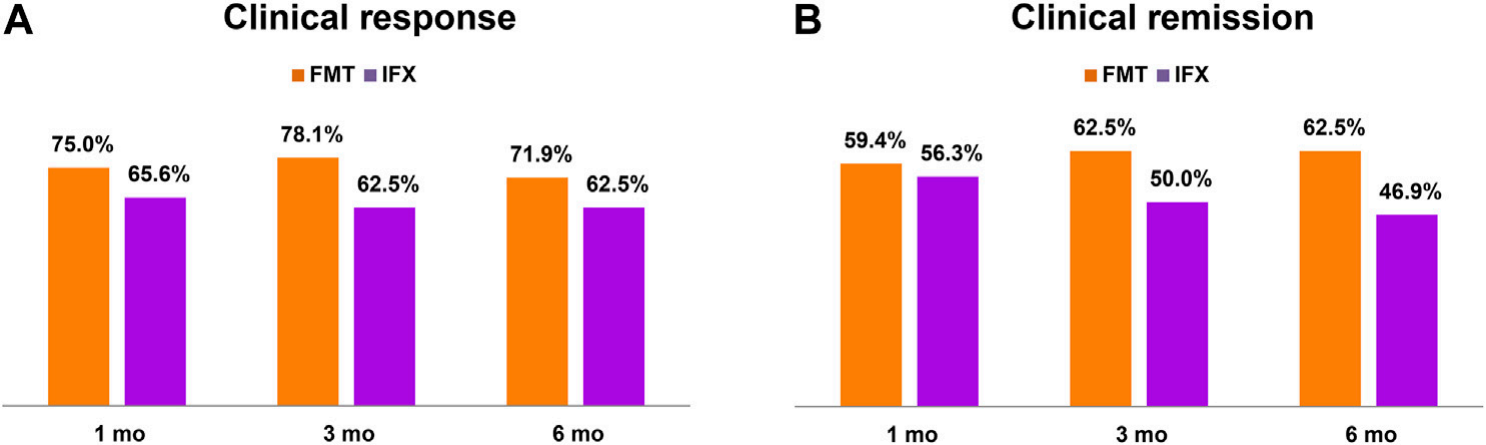
The efficacy of fecal microbiota transplantation (FMT) and infliximab (IFX).

Among the 32 patients, at one month, three months, and six months after FMT, the rates of clinical response were 75.0% (24/32), 78.1% (25/32) and 71.9% (23/32), respectively; and the clinical remission rates were 59.4% (19/32), 62.5% (20/32) and 62.5% (20/32), respectively (Figure 2). In the past treatment history, at one month, three months, and six months after IFX, the rates of clinical response were 65.6% (21/32), 62.5% (20/32) and 62.5% (20/32), respectively; and the clinical remission rates were 56.3% (18/32), 50.0% (16/32) and 46.9% (15/32), respectively. There was no apparent difference in efficacy at the three follow-up time points between IFX and FMT by Fisher’s exact test (FMT vs IFX, response: 1 month *p* = 0.585, 3 months *p* = 0.274, 6 months *p* = 0.595; remission: 1 month *p* = 1.000, 3 months *p* = 0.450, 6 months *p* = 0.315) (Figure 2).

**FIGURE 3:**
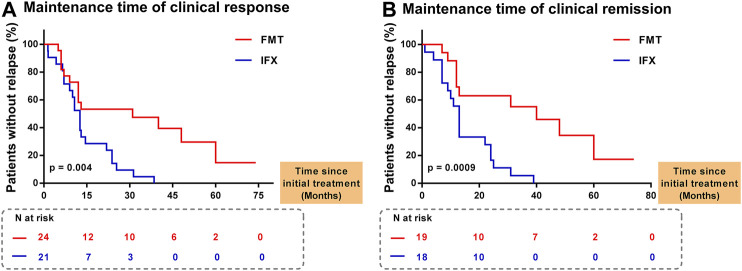
Evolution without clinical relapse **(A)** Time to lose response in patients who had achieved clinical response. At 1, 15, 30, 45, 60, and 75 months after FMT, 24, 12, 10, 6, 2 and 0 patients maintaining clinical response were followed, respectively. At 1, 15, 30, 45, 60, and 75 months after IFX, 21, 7, 3, 0, 0 and 0 patients maintaining clinical response were followed, respectively. Rates of clinical relapse were significantly higher in IFX treatment stage than those in FMT treatment stage (log-rank test *p* = 0.004) **(B)** Time to lose response in patients who had achieved clinical response. At 1, 20, 40, 60 and 80 months after FMT, 19, 10, 7, 2 and 0 patients maintaining clinical remission were followed, respectively. At 1, 20, 40, 60 and 80 months after IFX, 18, 10, 0, 0 and 0 patients maintaining clinical remission were followed, respectively. Rates of clinical relapse were significantly higher in IFX treatment stage than those in FMT treatment stage (log-rank test *p* = 0.0009). FMT, fecal microbiota transplantation; IFX, infliximab; N, number of patients.

As shown in Figure 3A, 21 patients achieved clinical response at one month after IFX, and 24 patients had clinical response at one month after FMT. Later, rates of clinical relapse were significantly higher in IFX treatment stage {log-rank test *p* = 0.004 (HR = 2.783 (95% confidence interval (CI) 1.446 to 5.355)). Similarly, in Figure 3B, rates of clinical relapse were significantly higher in IFX treatment stage (log-rank test *p* = 0.0009 [HR = 3.081 (95% CI 1.43–6.639)] in patients who reached clinical remission at one month after IFX (n = 18) and FMT (n = 19). The median maintaining time of the clinical remission from IFX was 13 months (IQR, 7–24), and from FMT was 17 months (IQR, 9–40). As shown in Figure 1, 10 patients achieving sustained clinical remission from FMT and without relapse at the final follow-up point, and their median maintaining time of clinical remission was 28 months (IQR, 7.75–42.5). However, all patients undergoing IFX treatment had disease relapse before being recruited into the current study.

### Clinical Factors Affecting Maintaining Time of Efficacy from FMT

To identify which clinical factors were correlated to the short-term and long-term efficacy of FMT, univariable and multivariable logistic regression analysis were used to evaluate independent predictors of efficacy at one month and one year after FMT.

In univariate analysis, the baseline HBI, disease activity, and with or without the second course of FMT before relapse significantly affected the short-term (one month) efficacy of FMT (*p* = 0.002, *p* = 0.003, *p* = 0.024). Patients with lower baseline HBI, mild disease activity or second course of FMT before relapse achieved remarkably better efficacy at one month after FMT. Additionally, the baseline HBI, disease activity and gender of donor were significantly associated with the long-term (one year) efficacy of FMT (*p* = 0.008, *p* = 0.005, *p* = 0.003) (Table 3). Lower baseline HBI, mild disease activity or microbiota suspension from female donors contributed to better clinical outcomes at one year after FMT.

**TABLE 3:** Clinical factors for efficacy of fecal microbiota transplantation.

N = 32	Remission (n = 19) (1 mo post-FMT)			Remission (n = 17) (1 yr post-FMT)		
Variables	Univariate	Multivariate		Univariate	Multivariate	
	*p*	Or (95%CI)	*p*	*p*	OR (95%CI)	*p*
Age at FMT	0.457	—	**–**	0.975	**–**	**–**
Gender	0.467	—	**–**	0.128	**–**	**–**
Age of diagnosis	0.975	—	**–**	0.338	**–**	**–**
Time from diagnosis to first FMT	0.312	—	**–**	0.574	**–**	**–**
Age of onset	0.365	—	**–**	0.626	**–**	**–**
Duration of disease	0.623	—	**–**	0.39	**–**	**–**
Time between final IFX and FMT	0.127	—	**–**	0.135	**–**	**–**
Reasons for the failure of IFX	0.961	—	**–**	0.712	**–**	**–**
Baseline HBI	0.002	0.888 (0.623–1.266)	0.512	0.008	0.850 (0.613–1.177)	0.328
Baseline disease activity	0.003	8.966 (0.414–194.39)	0.162	0.005	3.218 (0.162–63.79)	0.443
Disease location	0.735	—	—	0.178	—	—
Perianal disease	0.625	—	—	1.000	—	—
Delivering route	0.450	—	—	0.529	—	—
With EEN during FMT	0.598	—	—	0.430	—	—
With 2nd FMT before relapse	0.024	11.211 (0.794–158.21)	0.074	0.060	7.628 (0.303–191.89)	0.217
Form of bacterial suspension	1.000	—	—	1.000	—	—
Preparation of fecal microbiota	0.467	—	—	0.712	—	—
Age of donor	0.068	0.884 (0.733–1.066)	0.197	0.059	0.745 (0.533–1.042)	0.086
Gender of donor	0.473	—	—	0.003	0.027 (0.001–0.720)	0.031

Mo, month; yr, year; FMT, fecal microbiota transplantation; IFX, infliximab; HBI, Harvey-Bradshaw Index; EEN, exclusive enteral nutrition; *p* < 0.05 was considered statistically significant. Clinical factors with *p* < 0.1 by univariate analysis were included into multivariable logistic regression analysis.

Further multivariate analysis revealed that the gender of the donor (95% CI: 0.001–0.72; *p* = 0.031) was an independent predictor of efficacy at one year after FMT (Table 3). Patients undergoing FMT with microbiota suspension from female donors tended to have a higher possibility of achieving clinical remission at one year after FMT. As shown in Supplementary Figure S1, the area under the receiver operating characteristic curve of gender was 0.775 (95% CI: 0.603–0.946, *p* = 0.008), the sensitivity was 66.7%, the specificity was 88.2%.

### Safety

No serious AEs associated with FMT were observed during and after FMT. During the follow-up period, three episodes of AEs were observed in two patients, with the rate of AEs was 6.3% (2/32). All AEs occurred within 24 h after FMT, including fever and diarrhea, and were self-recovered without medication in the short term (Table 4).

**TABLE 4:** The adverse events of infliximab and fecal microbiota transplantation.

The AEs of IFX											
Pt	Related AEs	Time from the initial infusion		Time from the recent infusion		Causality	Discontinuation of IFX		SAEs		Cured
1	Fever	2 h		2 h		Probable	×		×		**√**
	Lymphatic *tuberculosis*	0.5 mo		0.5 mo		Probable	√		√		**√**
2	Rash	6.5 mo		2 h		Probable	×		×		**√**
	Dyspnea	7.5 mo		2 h		Probable	√		√		**√**
	Palpitation	7.5 mo		2 h		Probable	√		√		**√**
3	Laryngeal edema	0.5 mo		2 h		Probable	√		√		**√**
4	Pruritus	6.5 mo		2 h		Probable	×		×		**√**
5	Abdominal pain	2 h		2 h		Possible	×		×		**√**
	Loss of weight	1 mo		1 mo		Possible	√		√		**√**
6	Rash	2 h		2 h		Probable	×		×		**√**
	Intestinal bleeding	1 mo		1 mo		Possible	√		√		**√**
	Fatigue	1 mo		1 mo		Possible	×		×		**√**
7	Intestinal bleeding	24 h		24 h		Possible	×		×		**√**
8	Abnormal liver function	3 mo		0.5 mo		Probable	×		×		**√**
9	Chest distress	2 h		2 h		Probable	×		×		**√**
10	Laryngeal edema	7.5 mo		2 h		Probable	√		√		**√**
	Dyspnea	7.5 mo		2 h		Probable	√		√		**√**
11	Rash	2 h		2 h		Probable	×		×		**√**
	Tuberculosis	6 mo		1 mo		Probable	√		√		**√**
12	Fatigue	7.5 mo		1 mo		Possible	√		×		**√**
	Drowsiness	7.5 mo		1 mo		Possible	√		×		**√**
13	Flatulence	1 mo		1 mo		Possible	×		×		**√**
14	Fatigue	26 mo		2 mo		Possible	√		×		**√**
15	Rash	7.5 mo		2 h		Probable	√		×		**√**

The AEs of FMT											
Pt	AEs		Time from FMT		Causality			SAEs		Cured	
1	Fever		6 h		Probable			×		√	
2	Diarrhea		2 h		Probable			×		√	
	Fever		12 h		Probable			×		√	

AEs, adverse events; IFX, infliximab; SAEs, serious adverse events; FMT, fecal microbiota transplantation; hr, hour; mo, month.

During the follow-up of IFX treatment, 21.9% (7/32) of patients with serious AEs associated with IFX were observed, and 24 episodes of AEs were observed in 15 (15/32, 46.9%) patients. As listed in Table 4, AEs are mainly related to immunosuppression (infections) or drug-related immunogenicity (AIR and DHR). Two severe *tuberculosis* infections in two patients were recorded. Eleven patients had 14 episodes of AIR, including fever, rash, dyspnea, palpitation, laryngeal edema, pruritus, abdominal pain, intestinal bleeding and chest distress. In total, the occurrence of AIR led to IFX discontinuation in 4 patients. None of the AIR episodes was life-threatening. Eight patients had 10 episodes of DHR, including lymphatic *tuberculosis*, loss of weight, intestinal bleeding, fatigue, abnormal liver function, *tuberculosis*, drowsiness and flatulence, which lead to IFX discontinuation in 6 patients.

Comparing the incidences of AEs between FMT and IFX treatment by Fisher’s exact test, the rate of AEs from FMT was significantly lower than that from IFX (*p* = 0.002).

## Discussion

Loss of response to anti-TNF is frequent, estimated at 13% per year for IFX (Gisbert and Panes, 2009). In view of the present study, CD patients with prior loss of response or intolerance to IFX, switching to FMT that is effective and safe, seems to be a promising approach. Increasing studies have demonstrated that FMT could effectively induce clinical remission and improve clinical symptoms in patients with active CD in a short-term observation with a low incidence of AEs (Colman and Rubin, 2014; Cui et al., 2015a; Suskind et al., 2015; Sokol et al., 2020). The current real-world study evaluated the efficacy and safety of FMT for CD patients who had prior IFX failure and determined those clinical factors with the predictive role for the short-term and long-term therapeutic success or failure. Furthermore, our study first compared the efficacy and safety between FMT and IFX in the world.

Our remission rates at one month, three months and six months after FMT were higher than those of IFX without significant difference. The rates of clinical remission from FMT were about 60% within half a year after administration, which was consistent with those reported in the previous cohort and randomized controlled studies (Wang et al., 2018; Allegretti et al., 2020; Sokol et al., 2020; Xiang et al., 2020). In addition, our findings revealed that patients with prior loss of response or intolerance to IFX in CD could achieve a longer maintaining time of efficacy from FMT than that from the IFX treatment stage. Furthermore, similar to several previous reports, the results of the present study in Table 3 also demonstrated that patients undergoing multiple courses of FMT before relapse to consolidate efficacy could achieve longer maintaining time (Li et al., 2019; Sood et al., 2019; Li et al., 2020; Xiang et al., 2020).

In the multivariate analysis, the gender of the donor (female) was an independent factor for the efficacy of FMT, which indicated that patients undergoing FMT with microbiota suspension from female donors tended to have a higher possibility of achieving clinical remission at one year after FMT. Currently, the possible factors to affect the efficacy of FMT are focused on FMT methodology, including donor screening, laboratory preparation, and delivery ways (Lai et al., 2019). A meta-analysis review by Ianiro et al. reported that the colonoscopy was associated with higher efficacy rates of FMT for recurrent *Clostridium difficile* infection (rCDI) (Ianiro et al., 2018). Additionally, Sokol et al. first conducted a pilot randomized controlled study indicated that higher colonization by donor microbiota in patients with CD was associated with maintenance of remission in small sample sizes (Sokol et al., 2020). In our other studies with larger sample sizes (Li et al., 2019; Xiang et al., 2020), we did not find the independent factors for the efficacy of FMT in CD. The audience should be cautious with the present result on the gender of donors influencing the efficacy of FMT. More studies with larger sample sizes are necessary to confirm the factors that influence the efficacy of FMT.

The safety of FMT in CD has been reported by our recent work (Wang et al., 2018). Here, no long-term (>1 month) (Ding et al., 2019) FMT-related AEs and SAEs were observed during the follow-up. However, the rates of AEs and SAEs of IFX during the follow-up were significantly higher than those of FMT. Many AEs of IFX in our study led to IFX discontinuation, which is similar to the findings reported by Zabana et al. (Zabana et al., 2010). 34.4% (11/32) of patients had AIR during IFX treatment, but only 12.5% (4/32) had DHR in the study. The incidence of an AIR is about 6.5%, with mild, moderate, or severe reactions occurring in 3.1, 1.2, and 1% of IFX infusions, respectively (Cheifetz et al., 2003; Ma et al., 2019).

A study with a large sample size of Spanish demonstrated that the cumulative incidence of loss of response at 5 years was 45% after a second anti-TNF line and 38% after a third anti-TNF line (Casanova et al., 2020). Additionally, patients with immunogenic failure of a first anti-TNF easily developed failure of the second anti-TNF prescribed as monotherapy in 85% of patients within 2 years of follow-up (Roblin et al., 2018). Notably, according to the reports by Christian, biologicals are the main contributor to the cost of care for IBD from a European perspective, and clinicians need to be responsible for choosing therapies that are clinically safe and effective as well as affordable to society (Selinger, 2020).

The present study has several strengths: 1) With regard to FMT, all recipients had prior IFX failure; 2) Comparisons of efficacy and safety between FMT and IFX were performed in the longitudinal study, which is the first report so far in the world; 3) Our findings highlighted that FMT should be a new option for patients with prior IFX failure.

There were also some limitations in the present study. The clinical details of IFX were retrospective data from patients, which presented subjectivity. A scientific clinical predictive model should be established in further study on a larger sample size. A randomized and controlled study comparing the efficacy and safety between FMT and second or third line anti-TNF currently used by the patients with prior IFX failure should be conducted to draw a more definite conclusion.

In conclusion, compared with IFX, FMT had a similar effect of induction, longer maintaining time and a lower incidence of AEs in the present population. These results will encourage clinicians to consider FMT into practice as a new therapy option for patients with prior failure of IFX in CD.

## Data Availability

The original contributions presented in the study are included in the article/Supplementary Material, further inquiries can be directed to the corresponding author.

## Supplementary Material

Supplementary Figure S1 | Receiver operating characteristic curve (ROC) illustrating the performance of the predictive model. The area under the curve was 0.775 (95% confidence interval: 0.603–0.946).

## References

[B1] Allegretti J. R. Kelly C. R. Grinspan A. Mullish B. H. Hurtado J. Carrellas M. (2020). Inflammatory Bowel Disease Outcomes Following Fecal Microbiota Transplantation for Recurrent *C. difficile* Infection. Inflamm. Bowel Dis., izaa283. 10.1093/ibd/izaa283 33155639 PMC8376126

[B2] Allez M. Karmiris K. Louis E. Van Assche G. Ben-Horin S. Klein A. (2010). Report of the ECCO Pathogenesis Workshop on Anti-TNF Therapy Failures in Inflammatory Bowel Diseases: Definitions, Frequency and Pharmacological Aspects. J. Crohns. Colitis 4, 355–366. 10.1016/j.crohns.2010.04.004 21122530

[B3] Baumgart D. C. Sandborn W. J. (2007). Inflammatory Bowel Disease: Clinical Aspects and Established and Evolving Therapies. The Lancet 369, 1641–1657. 10.1016/S0140-6736(07)60751-X 17499606

[B4] Becker H. M. Grigat D. Ghosh S. Kaplan G. G. Dieleman L. Wine E. (2015). Living with Inflammatory Bowel Disease: A Crohn’s and Colitis Canada Survey. Can. J. Gastroenterol. Hepatol. 29, 77–84. 10.1155/2015/815820 25803017 PMC4373565

[B5] Billioud V. Sandborn W. J. Peyrin-Biroulet L. (2011). Loss of Response and Need for Adalimumab Dose Intensification in Crohn’s Disease: a Systematic Review. Am. J. Gastroenterol. 106, 674–684. 10.1038/ajg.2011.60 21407178

[B6] Buhl S. Steenholdt C. Rasmussen M. Borghede M. K. Brynskov J. Thomsen O. Ø. (2017). Outcomes after Primary Infliximab Treatment Failure in Inflammatory Bowel Disease. Inflamm. Bowel Dis. 23, 1210–1217. 10.1097/MIB.0000000000001117 28445244

[B7] Casanova M. J. Chaparro M. Mínguez M. Ricart E. Taxonera C. García-López S. (2020). Effectiveness and Safety of the Sequential Use of a Second and Third Anti-TNF Agent in Patients with Inflammatory Bowel Disease: Results from the Eneida Registry. Inflamm. Bowel Dis. 26, 606–616. 10.1093/ibd/izz192 31504569

[B8] Cheifetz A. Smedley M. Martin S. Reiter M. Leone G. Mayer L. (2003). The Incidence and Management of Infusion Reactions to Infliximab: a Large Center Experience. Am. J. Gastroenterol. 98, 1315–1324. 10.1111/j.1572-0241.2003.07457.x 12818276

[B9] Colman R. J. Rubin D. T. (2014). Fecal Microbiota Transplantation as Therapy for Inflammatory Bowel Disease: a Systematic Review and Meta-Analysis. J. Crohn’s Colitis 8, 1569–1581. 10.1016/j.crohns.2014.08.006a 25223604 PMC4296742

[B10] Colombel J. F. Sandborn W. J. Rutgeerts P. Enns R. Hanauer S. B. Panaccione R. (2007). Adalimumab for Maintenance of Clinical Response and Remission in Patients with Crohn’s Disease: the CHARM Trial. Gastroenterology 132, 52–65. 10.1053/j.gastro.2006.11.041 17241859

[B11] Cui B. Feng Q. Wang H. Wang M. Peng Z. Li P. (2015a). Fecal Microbiota Transplantation through Mid-gut for Refractory Crohn's Disease: Safety, Feasibility, and Efficacy Trial Results. J. Gastroenterol. Hepatol. 30, 51–58. 10.1111/jgh.12727 25168749

[B12] Cui B. Li P. Xu L. Zhao Y. Wang H. Peng Z. (2015b). Step-up Fecal Microbiota Transplantation Strategy: a Pilot Study for Steroid-dependent Ulcerative Colitis. J. Transl. Med. 13, 298. 10.1186/s12967-015-0646-2 26363929 PMC4567790

[B13] Ding X. Li Q. Li P. Zhang T. Cui B. Ji G. (2019). Long-Term Safety and Efficacy of Fecal Microbiota Transplant in Active Ulcerative Colitis. Drug Saf. 42, 869–880. 10.1007/s40264-019-00809-2 30972640

[B14] Fecal Microbiota Transplantation-standardization Study, G (2020). Nanjing Consensus on Methodology of Washed Microbiota Transplantation. Chin. Med. J. (Engl) 133, 2330–2332. 10.1097/CM9.0000000000000954 32701590 PMC7546843

[B15] Fine S. Papamichael K. Cheifetz A. S. (2019). Etiology and Management of Lack or Loss of Response to Anti-tumor Necrosis Factor Therapy in Patients with Inflammatory Bowel Disease. Gastroenterol. Hepatol. (N Y) 15, 656–665. 31892912 PMC6935028

[B16] Gagniere C. Beaugerie L. Pariente B. Seksik P. Amiot A. Abitbol V. (2015). Benefit of Infliximab Reintroduction after Successive Failure of Infliximab and Adalimumab in Crohn’s Disease. J. Crohn’s Colitis 9, 349–355. 10.1093/ecco-jcc/jju024 25547977

[B17] Gionchetti P. Dignass A. Danese S. Magro Dias F. J. Rogler G. Lakatos P. L. (2017). 3rd European Evidence-Based Consensus on the Diagnosis and Management of Crohn’s Disease 2016: Part 2: Surgical Management and Special Situations. Eccojc 11, 135–149. 10.1093/ecco-jcc/jjw169 27660342

[B18] Gisbert J. P. Panés J. (2009). Loss of Response and Requirement of Infliximab Dose Intensification in Crohn’s Disease: a Review. Am. J. Gastroenterol. 104, 760–767. 10.1038/ajg.2008.88 19174781

[B19] Hanauer S. B. Feagan B. G. Lichtenstein G. R. Mayer L. F. Schreiber S. Colombel J. F. (2002). Maintenance Infliximab for Crohn’s Disease: the ACCENT I Randomised Trial. The Lancet 359, 1541–1549. 10.1016/S0140-6736(02)08512-4 12047962

[B20] Ianiro G. Maida M. Burisch J. Simonelli C. Hold G. Ventimiglia M. (2018). Efficacy of Different Faecal Microbiota Transplantation Protocols for *Clostridium difficile* Infection: A Systematic Review and Meta‐analysis. United Eur. Gastroenterol. J. 6, 1232–1244. 10.1177/2050640618780762 PMC6169051 30288286

[B21] Kong L. Lloyd-Price J. Vatanen T. Seksik P. Beaugerie L. Simon T. (2020). Linking Strain Engraftment in Fecal Microbiota Transplantation with Maintenance of Remission in Crohn’s Disease. Gastroenterology 159, 2193–2202. 10.1053/j.gastro.2020.08.045 32860788 PMC7725862

[B22] Lai C. Y. Sung J. Cheng F. Tang W. Wong S. H. Chan P. K. S. (2019). Systematic Review with Meta-Analysis: Review of Donor Features, Procedures and Outcomes in 168 Clinical Studies of Faecal Microbiota Transplantation. Aliment. Pharmacol. Ther. 49, 354–363. 10.1111/apt.15116 30628108

[B23] Li P. Zhang T. Xiao Y. Tian L. Cui B. Ji G. (2019). Timing for the Second Fecal Microbiota Transplantation to Maintain the Long-Term Benefit from the First Treatment for Crohn’s Disease. Appl. Microbiol. Biotechnol. 103, 349–360. 10.1007/s00253-018-9447-x 30357440 PMC6311185

[B24] Li Q. Ding X. Liu K. Marcella C. Liu X. Zhang T. (2020). Fecal Microbiota Transplantation for Ulcerative Colitis: The Optimum Timing and Gut Microbiota as Predictors for Long-Term Clinical Outcomes. Clin. Translational Gastroenterol. 11, e00224, 10.14309/ctg.0000000000000224 PMC7431231 32955197

[B25] Li Q. Zhang T. Ding X. Xiang L. Cui B. Buch H. (2020). Enhancing Patient Adherence to Fecal Microbiota Transplantation Maintains the Long-Term Clinical Effects in Ulcerative Colitis. Eur. J. Gastroenterol. Hepatol. 32, 955–962. 10.1097/MEG.0000000000001725 32282545

[B26] Long C. Yu Y. Cui B. Jagessar S. A. R. Zhang J. Ji G. (2018). A Novel Quick Transendoscopic Enteral Tubing in Mid-gut: Technique and Training with Video. BMC Gastroenterol. 18, 37. 10.1186/s12876-018-0766-2 29534703 PMC5850973

[B27] Ma D. Wong W. Aviado J. Rodriguez C. Wu H. (2019). Safety and Tolerability of Accelerated Infliximab Infusions in Patients with Inflammatory Bowel Disease. Am. J. Gastroenterol. 114, 352–354. 10.1038/s41395-018-0368-1 30333541

[B28] Papamichael K. Cheifetz A. S. Irving P. M. (2020). New Role for Azathioprine in Case of Switching Anti-TNFs in IBD. Gut 69, 1165–1167. 10.1136/gutjnl-2020-320677 32075889

[B29] Papamichael K. Gils A. Rutgeerts P. Levesque B. G. Vermeire S. Sandborn W. J. (2015). Role for Therapeutic Drug Monitoring during Induction Therapy with TNF Antagonists in IBD. Inflamm. Bowel Dis. 21, 182–197. 10.1097/MIB.0000000000000202 25222660

[B30] Peng Z. Xiang J. He Z. Zhang T. Xu L. Cui B. (2016). Colonic Transendoscopic Enteral Tubing: A Novel Way of Transplanting Fecal Microbiota. Endosc. Int. Open 04, E610–E613. 10.1055/s-0042-105205 PMC4993903 27556065

[B31] Roblin X. Vérot C. Paul S. Duru G. Williet N. Boschetti G. (2018). Is the Pharmacokinetic Profile of a First Anti-TNF Predictive of the Clinical Outcome and Pharmacokinetics of a Second Anti-TNF? Bowel Dis. 24, 2078–2085. 10.1093/ibd/izy111 29718216

[B32] Roblin X. Williet N. Boschetti G. Phelip J.-M. Del Tedesco E. Berger A.-E. (2020). Addition of Azathioprine to the Switch of Anti-TNF in Patients with IBD in Clinical Relapse with Undetectable Anti-TNF Trough Levels and Antidrug Antibodies: a Prospective Randomised Trial. Gut 69, 1206–1212. 10.1136/gutjnl-2019-319758 31980448

[B33] Selinger C. P. (2020). Biologicals Are the Main Contributor to Cost of Care for IBD: a European Perspective. Lancet Gastroenterol. Hepatol. 5, 421–422. 10.1016/S2468-1253(20)30042-X 32061323

[B34] Sokol H. Landman C. Landman C. Seksik P. Berard L. Montil M. (2020). Fecal Microbiota Transplantation to Maintain Remission in Crohn’s Disease: a Pilot Randomized Controlled Study. Microbiome 8, 12. 10.1186/s40168-020-0792-5 32014035 PMC6998149

[B35] Sood A. Mahajan R. Singh A. Midha V. Mehta V. Narang V. (2019). Role of Faecal Microbiota Transplantation for Maintenance of Remission in Patients with Ulcerative Colitis: A Pilot Study. J. Crohns. Colitis. 13, 1311–1317. 10.1093/ecco-jcc/jjz060 30873549

[B36] Suskind D. L. Brittnacher M. J. Wahbeh G. Shaffer M. L. Hayden H. S. Qin X. (2015). Fecal Microbial Transplant Effect on Clinical Outcomes and Fecal Microbiome in Active Crohnʼs Disease. Inflamm. Bowel Dis. 21, 556–563. 10.1097/MIB.0000000000000307 25647155 PMC4329080

[B37] Terdiman J. P. Gruss C. B. Heidelbaugh J. J. Sultan S. Falck–Ytter Y. T. Practice A. G. A. I. C. (2013). American Gastroenterological Association Institute Guideline on the Use of Thiopurines, Methotrexate, and Anti-TNF-α Biologic Drugs for the Induction and Maintenance of Remission in Inflammatory Crohn’s Disease. Gastroenterology 145, 1459–1463. 10.1053/j.gastro.2013.10.047 24267474

[B38] Thia K. T. Sandborn W. J. Harmsen W. S. Zinsmeister A. R. Loftus E. V. Jr. (2010). Risk Factors Associated with Progression to Intestinal Complications of Crohn’s Disease in a Population-Based Cohort. Gastroenterology 139, 1147–1155. 10.1053/j.gastro.2010.06.070 20637205 PMC2950117

[B39] Wang H. Cui B. Li Q. Ding X. Li P. Zhang T. (2018). The Safety of Fecal Microbiota Transplantation for Crohn’s Disease: Findings from A Long-Term Study. Adv. Ther. 35, 1935–1944. 10.1007/s12325-018-0800-3 30328062 PMC6223988

[B40] Xiang L. Ding X. Li Q. Wu X. Dai M. Long C. (2020). Efficacy of Faecal Microbiota Transplantation in Crohn’s Disease: a New Target Treatment? Microb. Biotechnol. 13, 760–769. 10.1111/1751-7915.13536 31958884 PMC7111085

[B41] Zabana Y. Domènech E. Mañosa M. Garcia-Planella E. Bernal I. Cabré E. (2010). Infliximab Safety Profile and Long-Term Applicability in Inflammatory Bowel Disease: 9-year Experience in Clinical Practice. Aliment. Pharmacol. Ther. 31, 553–560. 10.1111/j.1365-2036.2009.04206.x 20002026

[B42] Zhang F. Cui B. Cui B. He X. Nie Y. Wu K. (2018). Microbiota Transplantation: Concept, Methodology and Strategy for its Modernization. Protein Cell 9, 462–473. 10.1007/s13238-018-0541-8 29691757 PMC5960466

[B43] Zhang T. Lu G. Zhao Z. Liu Y. Shen Q. Li P. (2020). Washed Microbiota Transplantation vs. Manual Fecal Microbiota Transplantation: Clinical Findings, Animal Studies and *in vitro* Screening. Protein Cell 11, 251–266. 10.1007/s13238-019-00684-8 31919742 PMC7093410

