# Supplementary figures and images for: Fecal Microbiota Transplantation is a Promising Switch Therapy for Patients with Prior Failure of Infliximab in Crohn’s Disease
Source: Front Pharmacol. 2021 May 17;12:658087. doi: 10.3389/fphar.2021.658087 (PMC8166050; doi:10.3389/fphar.2021.658087)

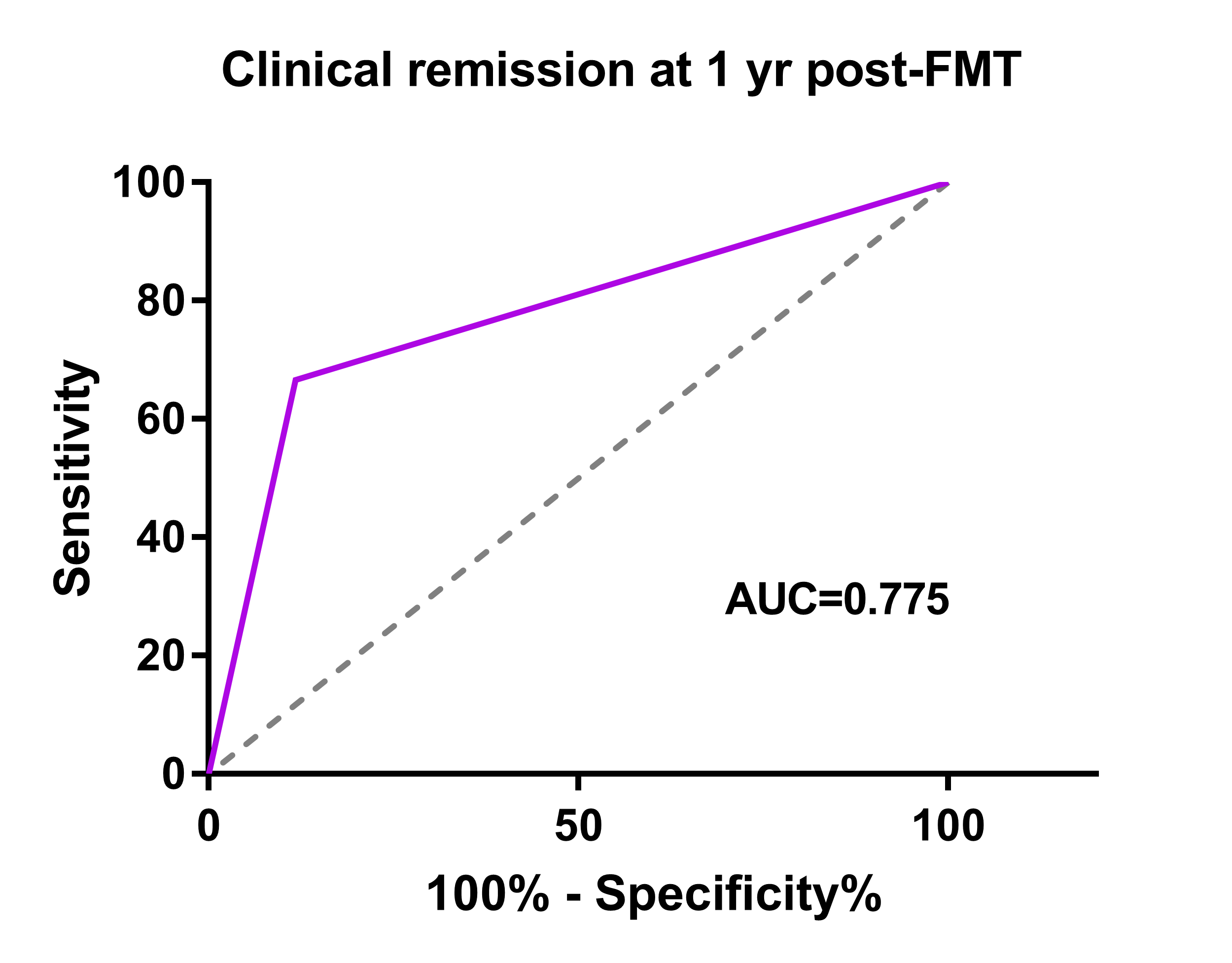

Supplement: Supplementary file 1 [file Image1.TIF]
